# Helminth and Intestinal Protozoa Infections, Multiparasitism and Risk Factors in Champasack Province, Lao People's Democratic Republic

**DOI:** 10.1371/journal.pntd.0001037

**Published:** 2011-04-12

**Authors:** Somphou Sayasone, Tippi K. Mak, Monely Vanmany, Oroth Rasphone, Penelope Vounatsou, Jürg Utzinger, Kongsap Akkhavong, Peter Odermatt

**Affiliations:** 1 National Institute of Public Health, Ministry of Health, Vientiane, Lao People's Democratic Republic; 2 Department of Epidemiology and Public Health, Swiss Tropical and Public Health Institute, Basel, Switzerland; 3 University of Basel, Basel, Switzerland; 4 Department of Radiology, Mahosot Hospital, Ministry of Health, Vientiane, Lao People's Democratic Republic; George Washington University, United States of America

## Abstract

**Background:**

Detailed investigations of multiparasitism are scarce in the Mekong River basin. We assessed helminth (trematode, nematode, and cestode), and intestinal protozoa infections, and multiparasitism in random population samples from three different eco-epidemiological settings in Champasack province, southern Lao People's Democratic Republic (Lao PDR), and determined underlying risk factors.

**Methodology:**

Two stool samples were collected from 669 individuals aged ≥6 months over consecutive days and examined for helminth infections using the Kato-Katz method. Additionally, one stool sample per person was subjected to a formalin-ethyl acetate concentration technique for diagnosis of helminth and intestinal protozoa infections. Questionnaires were administered to obtain individual and household-level data pertaining to behavior, demography and socioeconomic status. Risk factors for hepato-biliary and intestinal parasitic infections and multiparasitism were determined using multiple logistic regressions analyses.

**Principal Findings:**

Multiple species intestinal parasite infections were common: 86.6% of the study participants harbored at least two and up to seven different parasites concurrently. Regarding nematode infections, hookworm was the most prevalent species (76.8%), followed by *Ascaris lumbricoides* (31.7%) and *Trichuris trichiura* (25.0%). Regarding trematodes, *Opisthorchis viverrini* and *Schistosoma mekongi* infections were found in 64.3% and 24.2% of the participants, respectively. Infections with intestinal protozoa were rare.

**Conclusions/Significance:**

There is a pressing need to intensify and sustain helminth control interventions in the southern part of Lao PDR. Given the high prevalence with nematode and trematode infections and the extent of multiparasitism, preventive chemotherapy is warranted. This intervention should be coupled with health education and improved access to clean water and adequate sanitation to consolidate morbidity control and enhance sustainability.

## Author Summary

Multiparsitism is a general public health concern in tropical countries, and is of particular importance in the Mekong River basin of Southeast Asia. Here, we report results obtained from an in-depth study of hepato-biliary and intestinal multiparasitism and associated risk factors in three settings of the most southern province of Lao People's Democratic Republic. Multiple species intestinal parasite infections were very common: more than 80% of the study participants harbored at least two and up to seven different intestinal parasites concurrently. Of particular concerns are the high prevalence of the liver fluke *Opisthorchis viverrini* (64.1%) and the moderate prevalence of the blood fluke *Schistosoma mekongi* (24.2%), as these fluke infections are responsible for severe hepato-biliary morbidity, including the bile duct cancer cholangiocarcinoma. Hookworm was the most common nematode infection (76.8%). We conclude that given the very high prevalence rates of parasite infections and the extent of multiparasitism, regular deworming is warranted and that this intervention should be coupled with health education and improved assess to clean water and adequate sanitation to consolidate morbidity control and ensure long-term sustainability.

## Introduction

Lao People's Democratic Republic (Lao PDR) is a landlocked country situated in the Great Mekong sub-region of Southeast Asia, where socioeconomic and eco-epidemiological characteristics vary greatly according to location. In the northern part similar ecosystems are found as in southern People's Republic of China (P.R. China) with mountains and highlands dominating the landscapes. These topological features are natural barriers that might impede social and economic development, since transportation of commodities, communication and other exchanges are hampered. These issues exacerbate people's access to health care, clean water and adequate sanitation. Indeed, according to the results of the national population and housing census carried out in 2005, less than 20% and only about half of the population living in these areas had access to clean water and sanitation, respectively [1]. Water supply and sanitation are intimately linked with intestinal parasitic infections and poverty. Schistosomiasis, opisthorchiasis and infections with the common soil-transmitted helminths (i.e. *Ascaris lumbricoides*, hookworm, and *Trichuris trichiura*) are of particular relevance [2]–[5]. Improving socioeconomic status, including enhanced access to quality health care, safe water, and adequate sanitation have the potential to significantly reducing the prevalence and intensity of parasitic infections, and hence reduce disease-related morbidity [5]–[7].

The central and southern parts of Lao PDR are the low land along the Mekong River basin. In these regions, the socioeconomic conditions and means of communication and transport are more advanced than in northern Lao PDR. In recent years, through the formation of the ASEAN community, the economy of the Great Mekong sub-regions countries has been bolstered. Along with these changes and ecological transformations (e.g., deforestation and water resources developments), particularly in the lowlands of the Mekong River basin, patterns of parasitic infections are changing [8]. A matter of considerable public health concern is the transmission of *Schistosoma mekongi* which, although several rounds of preventive chemotherapy were implemented, is still transmitted in the Mekong River in the most southern province of Lao PDR [9], [10]. Furthermore, high prevalences of *Opisthorchis viverrini* are a concern, as this liver fluke is the main risk factor for the fatal cholangiocarcinoma bile duct cancer [11]–[13]. An infection with *O. viverrini* is acquired through the consumption of traditional dishes (e.g., “Lap-pa” and “Koy-pa”) prepared with raw or insufficiently cooked fish [14], [15]. The habit of eating raw or undercooked freshwater fish and other aquatic products is also a risk for acquiring small intestinal trematode infections, such as heterophyid and lecithodendriid flukes, which are endemic in southern Lao PDR [16]–[18]. Moreover, raw fish consumption is a precondition of capillariasis transmission, which was recently documented in Lao PDR [19], [20]. A national survey conducted among school children found that common soil-transmitted helminth infections are particularly prevalent in the northern provinces, whereas *O. viverrini* infections are rampant in the central and southern provinces with significant overlaps of different parasite species in all provinces [21]. It follows that multiparasitism must occur, which has been confirmed in recent surveys [15], [16], [22]. However, data pertaining to multiparasitism have mostly been obtained from small studies (e.g., in a single community in a single village), often looking at a narrow age range (e.g., school-aged children) [23].

The present study was carried out in different eco-epidemiological settings of Champasack province, southern Lao PDR. Using a cross-sectional design, the purpose was to assess the prevalence and intensity of hepato-biliary and intestinal parasitic infections and intestinal multiparasitism, and to identify underlying risk factors.

## Methods

### Study area

**Figure 1:**
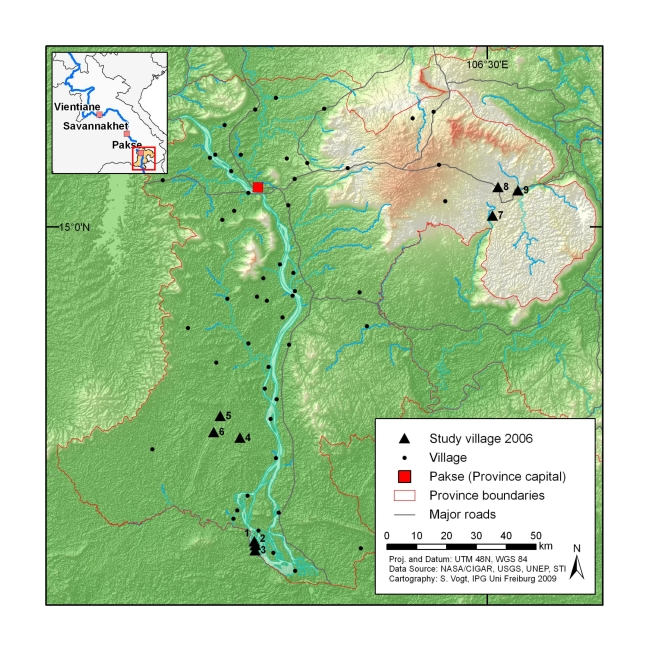
Map of Champasack province in southern Lao PDR with location of study villages sampled in early 2006.

The study was carried out in three distinct eco-epidemiological settings of Champasack province (Figure 1), located in the southern part of Lao PDR, namely (i) Khong, (ii) Mounlapamok, and (iii) Paksong districts. Of note, the districts represent different settings in terms of socioeconomic conditions and eco-epidemiology and are characteristic for other parts of Lao PDR.

Khong district (estimated population: 80,000) [24] is an island district which is located in the southern part of the province (∼120 km from Pakse city), which borders Cambodia (geographical coordinates: 13.57°–14.14°N latitude and 105.44°–106.08°E longitude). Khong district comprises dozens of islands in the Mekong River basin and is therefore also known as “district of four thousand islands”. The waterfall ‘Khon-Phapheng’ has put a barrier in the Mekong River and has created a natural reservoir. The ecology of the area is suitable for aquatic snails, the intermediate hosts for *S. mekongi* and food-borne trematodes, such as *O. viverrini* and minute intestinal flukes (MIF).

The Mounlapamok district is also located in the southern part of the province (∼80 km from Pakse city) with an estimated population of 40,000 [24]. It is a lowland district situated along the Mekong River (geographical coordinates: 14.15°–14.25°N and 105.49°–106.11°E). In this area, opisthorchiasis is highly prevalent [25].

Paksong district is located on the Bolovan plateau (geographical coordinates: 14.58°–15.23° N and 105.55°–106.48° E) at an elevation of ∼1,000 m above sea level in the northeast of the province (∼50 km from Pakse city). It is a mountainous area with an estimated population of 65,000 [24]. Soil-transmitted helminth infections are common in Paksong district [21].

### Ethical consideration and treatment

The study was approved by the Ethics Committee in Basel, Switzerland (EKBB; reference no. 255/06) and the National Ethics Committee, Ministry of Health (MoH) in Vientiane, Lao PDR (reference no. 027/NECHR). Permission for field work was obtained from MoH, the Provincial Health Office (PHO) and the District Health Office (DHO). Village meetings were held and village authorities and villagers were given detailed explanations about the aims, procedures, potential risks and benefit of the study. An information sheet in the local language was read aloud to all household members and their questions answered. Individual oral consent was obtained from all adult household members (literacy is very low in this part of Lao PDR, and hence we opted for oral rather than written consent). However, written informed consent was obtained from all heads of households. A witness observing this procedure also signed the consent form.

All individuals infected with *O. viverrini*, *S. mekongi*, soil-transmitted helminths, and intestinal protozoa were treated according to national guidelines [26]. An anti-spasmodic treatment and oral rehydration was provided in case of adverse events following drug administration.

### Study design and population surveyed

Our cross-sectional surveys were carried out between March and May 2006. In each setting, three villages were selected from the available village list in collaboration with the DHO, and 20–25 households were randomly selected in each village. All household members aged ≥6 months were invited to participate. The number of inhabitants per household was recorded. Unique identifiers were assigned to households and study participants.

### Field and laboratory procedures

In each village, a house (usually a school or a temple) was designated as an area of work for Kato-Katz (KK) thick smear preparation, microscopic examination of stool samples, etc. Two members of our research team (one interviewer and one general physician) went from house to house and interviewed first the head of household and then the remaining household members. Two questionnaires were administrated in each household. The household questionnaire (after pre-testing in a neighboring area) was administered to the heads of household. Data pertaining to household characteristics (e.g., building type and water supply), asset ownership (e.g., farm engine and bicycle) and ownership of animals (e.g., buffalo and cow) were collected. The geographical coordinates of each household were obtained by using a hand-held global positioning system (GPS) receiver (Garmin Ltd., Olathe, USA). Next, a pre-tested individual questionnaire was used and all household members were interviewed for demographic data (e.g., age, sex, educational attainment, and professional activity) and behavioral risks (e.g., food consumption habits and personal hygiene). Parents or legal caregivers answered for children.

Finally, stool containers were prepared for all members of each study household. Participants' names and unique identifiers were marked on the containers and distributed to the heads of household with detailed instructions of how to collect a fresh morning stool sample. All study participants were asked to provide a sufficiently large stool sample (at least 5 g) so that both KK and the formalin-ethyl acetate concentration technique (FECT) could be performed. After filled containers were collected, new empty containers were handed out with the goal to obtain three stool samples from each participant over consecutive days.

Stool samples were processed in the designated area of work in the study village within a maximum of 2 hours after collection by experienced laboratory technicians. A single KK thick smear was prepared from each stool sample, using a standard plastic template holding 41.7 mg of stool [27]. Slides were allowed to clear for 30 min prior to examination under a microscope. The number of eggs was counted and recorded for each helminth species separately.

Additionally, exactly 300 mg of stool taken from one sample was fixed in a tube containing 10 ml of sodium acetate acetic-acid formalin (SAF) [28]. SAF-fixed samples were forwarded to the parasitological department of the Faculty of Medicine, National University of Lao PDR. The samples were subjected to FECT [29] and diagnosed for the presence of intestinal protozoa and helminth species-specific infections and intensities with the assistance of laboratory staff from the Swiss Tropical and Public Health Institute (Basel, Switzerland).

### Statistical analysis

Data were double-entered and cross-checked using EpiData version 3.1 (Epidata Association; Odense, Denmark). Statistical analyses were performed with STATA version 10 (Stata Corporation; College Station, TX, USA). Only those individuals who had at least two KK thick smear readings and an additional FECT result, and complete questionnaire data were included in the final analyses.

People's socioeconomic status was estimated using a household-based asset approach and the population was stratified into wealth quintiles, namely (i) poorest, (ii) very poor, (iii) poor, (iv) less poor, and (v) least poor. Wealth quintiles were constructed using principal component analysis (PCA), as proposed by the Health Nutrition and Population/World Bank in 2000 [30]. Details of this widely used approach have been presented elsewhere [31]. In brief, a PCA was calculated from the following household assets: electricity radio/recorder, television, CD/DVD player, water pump, refrigerator, car, farm engine, motorcycle, rice security, house characteristics (construction material for floor, wall, and roof), and animal ownership (buffalo, cow, goat, and pig). The weights obtained from the first dimension were used to calculate the household index score. The first principal component (PC) explained 17.2% of the total variability. The greatest weights were attached to families living in a wooden house (0.30), a bamboo house (0.29), and the presence of a television at home (0.20). After standardization of these weighted asset variables, families living in a cement house had the highest scores (0.47). Lowest scores were attached to families living in a bamboo house (−0.55). The sum of total asset scores was assigned to each study participant.

Point prevalence of parasitic infections were determined and stratified by study area, sex, and age group. A chi-square (χ2) test was employed to investigate associations between categorical variables (e.g., between infection status and sex, age group, and study area). Study participants were subdivided into five age groups, namely (i) <5 years, (ii) 6–15 years, (iii) 16–30 years, (iv) 31–55 years, and (v) >55 years. The intensity of helminth egg counts was expressed as eggs per gram of stool (EPG). Intensity rate ratio (IRR) of EPG was calculated using negative binomial regression models and associated with sex and age groups. A predictor variable with level of significance below 0.15 in the bivariate logistic regression models was included in the multiple logistic regressions to investigate the associations between the parasitic infections and a particular risk factor. Random effect models were fitted into all regressions, taking into account the random effect of households.

## Results

### Study cohort and socioeconomic profile

**Figure 2:**
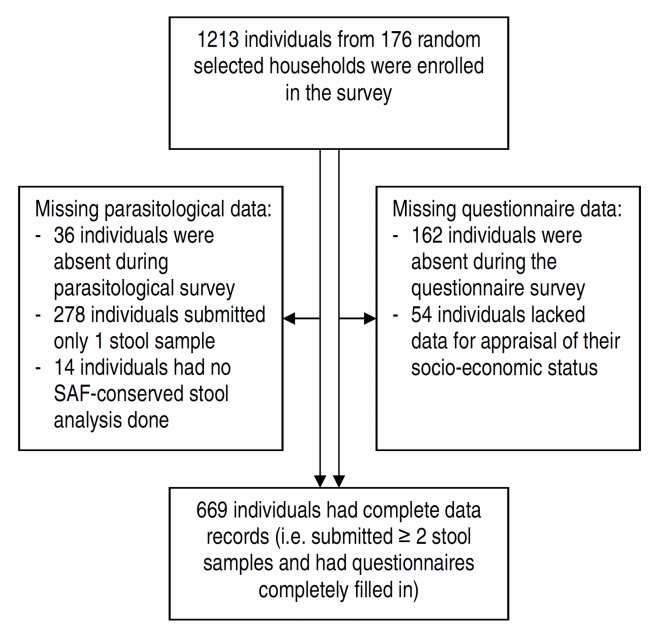
Study participants' compliance of survey in three eco-epidemiological settings of Champasack, southern Lao PDR.

From 1,213 enrolled participants, 1,051 were present during the cross-sectional survey and responded to our questionnaire (Figure 2). A total of 314 individuals (29.9%) failed to submit sufficient numbers and/or quantities of stool samples for laboratory diagnoses. Fourteen individuals (1.3%) had no SAF-fixed stool sample and 192 individuals (18.3%) were absent during the household-based interviews, and hence their socioeconomic status could not be determined. Overall, 669 individuals (63.7%) had complete data records (i.e., at least 2 KK thick smears, 1 FECT result, and complete questionnaire data).

Among this cohort, 212 individuals (31.7%) were from Paksong district, 232 (34.7%) from Mounlapamok district, and 225 (33.6%) from Khong district. Most study participants belonged to the Lao-loum ethnic groups (68.5%), whereas the Lao-theung minority accounted for the remaining 31.5%. There were slightly more females (n = 347, 51.9%). The median age was 15 years (range: 6 months to 87 years). Age structure was as follows: ≤5 years (17.3%), 6–15 years (32.9%), 16–30 years (16.4%), 31–55 years (24.9%) and >55 years (8.4%). Adults were primarily engaged in subsistence farming (52.1%), while there were only few government employees (1.4%). No professional activity accounted for 46.5% of the study participants. Of those, 17.3%, 20.4% and 8.8% were preschool-aged children, pupils or students and elderly persons, respectively.

**Figure 3:**
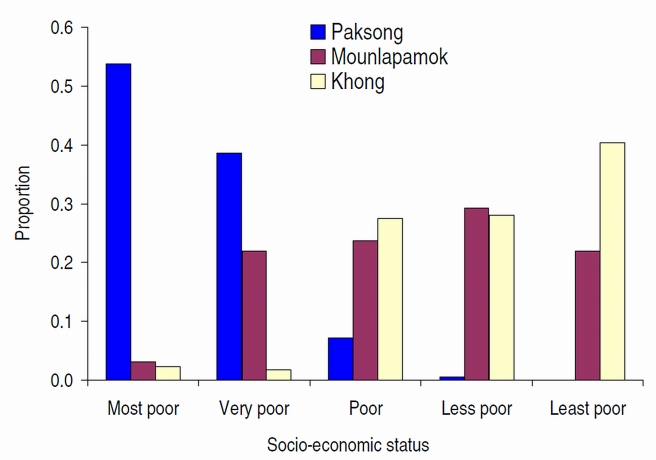
Socioeconomic status among individuals from Champasack province, southern Lao PDR, stratified by study setting (n = 669).

With regard to wealth, we observed that most study participants from Paksong district belonged to the poorest group (53.5%), whereas none of them were classified into the group of the least poor. In Khong and Mounlapamok districts, the combined percentage of less poor and least poor was 40.4% and 29.3%, respectively. Only a few individuals (Mounlapamok: 3.0% and Khong: 2.2%) belonged to the poorest group (Figure 3).

### Helminth and intestinal protozoa infections

Table 1 summarizes the results from the cross-sectional parasitological surveys, stratified by eco-epidemiological setting, sex, and age group. Analysis of at least two stool samples using the K-K technique, supplemented with an additional FECT result revealed overall infection prevalences of *O. viverrini*, *S. mekongi* and *Echinostoma* spp. of 64.3%, 24.2% and 6.0%, respectively. The former two trematode infections were particularly prevalent in Khong district (*O. viverrini*: 92.0%, *S. mekongi*: 68.0%). While a similarly high prevalence of *O. viverrini* infection was observed in Mounlapamok district (90.9%), the observed prevalence of *S. mekongi* was only 3.9%. In Paksong district only few cases of *O. viverrini* infections were observed (5.7%), owing to a highly significant difference among study location (likelihood ratio test (LRT)  = 51.35, P <0.001). The prevalence of *O. viverrini* infections increased with age and reached the highest levels in the age group above 55 years (LRT = 28.83, P <0.001). *S. mekongi* infections were also significantly associated with age, with the highest prevalence observed in the oldest age group (32.1%; LRT = 13.91, P = 0.007). Neither *O. viverrini* nor *S. mekongi* infections were significantly associated with sex. The prevalence of *O. viverrini* infections was significantly higher in Lao-loum ethnic group compared to Lao-theung (91.1% *vs.* 6.2%; LRT = 199.51, P <0.001).

**Table 1:** Prevalence of intestinal parasitic infections diagnosed by Kato-Katz plus formalin-ethyl-acetate concentration (n = 669).

Parasites	Prevalence (95% CI)	Study settings			Sex		Age groups (years)				
	(n = 669)	Khong (n = 225)	Mounlapamok (n = 232)	Paksong (n = 212)	Female (n = 347)	Male (n = 322)	≤5 (n = 116)	6–15 (n = 220)	16–30 (n = 110)	31–55 (n = 167)	>55 (n = 56)
**Trematodes**											
*O. viverrini*	64.3 (60.6–67.9)	92.0	90.9	5.7**	65.1	63.4	44.0	64.6	69.1	70.1	78.6
*S. mekongi*	24.2 (21.0–27.5)	68.0	3.9	0.0**	24.2	24.2	9.9	30.0	15.4	22.2	32.1
*Echinostoma* spp.	6.0 (4.2–7.8)	12.9	4.7	0.0**	6.1	5.9	3.5	4.6	4.6	5.4	5.4
**Nematodes**											
Hookworm	76.8 (73.6–80.0)	71.1	66.0	94.8**	76.7	77.0	39.7	80.0	31.8	78.4	75.0
*A. lumbricoides*	31.7 (28.2–35.2)	7.1	6.0	85.9**	31.7	31.7	39.7	31.8	31.8	29.9	23.2
*T. trichiura*	25.0 (21.7–28.3)	13.3	8.2	55.7**	23.6	26.4	19.0	26.4	26.4	25.2	14.3
*S. stercoralis*	4.6 (3.0–6.0)	9.8	3.9	0.0**	14.9	4.4	4.3	1.8	1.8	7.8	5.4
*E. vermicularis*	3.6 (2.2–5.0)	3.1	4.4	3.3	5.2	1.9*	2.6	2.7	3.6	3.6	8.9
**Cestodes**											
*Taenia* spp.	3.7 (2.3–5.2)	1.8	4.3	5.2	3.1	4.7	0.9	2.7	3.6	6.0	7.1
*H. diminuta*	2.7 (1.5–3.9)	0.0	0.0	8.5**	2.9	2.5	1.7	2.3	2.7	3.6	3.6
*D. latum*	0.5 (<0.1–0.9)	0.5	0.9	0.0	0.6	0.3	0.9	0.5	0.9	0.0	0.0
**Intestinal protozoa**											
*B. hominis*	13.6 (11.0–16.2)	19.6	6.5	15.1**	13.0	14.3	7.8	15.0	13.6	15.0	16.1
*E. coli*	7.2 (5.2–9.1)	3.0	8.4	10.4**	7.8	6.5	6.0	7.3	9.1	4.8	12.5
*G. intestinalis*	4.9 (3.3–6.6)	3.0	5.9	6.1	2.6	7.6*	5.2	6.4	8.2	1.8	1.8
*E. nana*	0.6 (<0.1–1.2)	0.6	0.4	0.5	0.3	0.9	0.9	0.9	0.0	0.6	0.0

CI, confidence interval.

*P-value <0.05;

**P-value <0.001.

*P-*value based on likelihood ratio test

The overall infection prevalence of hookworm, *A. lumbricoides* and *T. trichiura* was 76.8%, 31.7% and 25.0%, respectively. There was significant variation from one district to another (LRT = 30.11, P <0.001). The highest prevalences were found in Paksong district (hookworm: 94.8%, *A. lumbricoides*: 85.9%, and *T. trichiura*: 55.7%) and the lowest prevalences were observed in Mounlapamok district (hookworm: 66.0%, *T. trichiura*: 8.2%, and *A. lumbricoides*: 6.0%). There were no significant differences between sex and age groups for any of the three main soil-transmitted helminth infections.

Cestode infections such as *Taenia* spp., *Hymenolepis diminuta* and *Diphyllobothrium latum* were found at low prevalences, ranging between 0.5% and 3.7%. *Blastocystis hominis* (13.6%) was the most common intestinal protozoa diagnosed, followed by *Entamoeba coli* (7.2%), *Giardia intestinalis* (4.9%), and *Endolimax nana* (0.6%). There was a significant variation in the observed prevalence (LRT = 42.32, P<0.001) for *B. hominis* and *E. coli* according to study location.

### Infection intensities and multiparasitism

Table 2 shows the adjusted IRR of helminth egg counts expressed in EPG for the most prevalent intestinal parasites investigated, using age group <5 years as a referent group. The overall intensity ratio of EPG for *O. viverrini* infection increased with age and reached the highest level in the adult people aged above 55 years (IRR = 7.41, 95% confidence interval (CI)  = 4.82–11.42) with no significant sex difference. Children (6–15 years) showed a higher infection intensity with *S. mekongi* (IRR = 1.79, 95% CI = 1.01–3.18) and hookworm (IRR = 1.49, 95% CI = 1.17–1.90) than their older counterparts. With regard to *A. lumbricoides* and *T. trichiura*, there was no significant difference for infection intensity in all age groups.

**Table 2:** Negative binomial regression analyses for parasite eggs count in Champasack province (n = 669).

	*O. viverrini*		*S. mekongi*		Hookworm		*A. lumbricoides*		*T. trichiura*	
	IRR (95% CI)	*P*-value	IRR (95% CI)	*P*-value	IRR (95% CI)	*P*-value	IRR (95% CI)	*P*-value	IRR (95% CI)	*P*-value
**Sex**										
Female	1.00		1.00		1.00		1.00		1.00	
Male	0.92 (0.76–1.11)	0.404	1.18 (0.85–1.64)	0.308	1.08 (0.93–1.25)	0.321	0.96 (0.81–1.12)	0.593	0.91 (0.72–1.16)	0.437
**Age groups (years)**										
≤ 5	1.00		1.00		1.00		1.00		1.00	
6–15	2.95 (2.08–4.19)		1.79 (1.01–3.18)		1.49 (1.17–1.90)		1.25 (0.98–1.61)		1.10 (0.74––1.64)	
16–30	5.37 (3.68–7.84)		1.39 (0.72–2.68)		1.30 (0.99–1.69)		0.85 (0.63–1.13)		0.98 (0.63–1.54)	
31–55	7.18 (5.01–10.29)		1.06 (0.57–1.96)		1.16 (0.90–1.50)		1.09 (0.84–1.42)		0.88 (0.58–1.34)	
>55	7.41 (4.82–11.42)	<0.001	1.30 (0.63–2.67)	0.102	1.19 (0.85–1.67)	0.008	0.81 (0.55–1.19)	0.170	1.29 (0.66–2.53)	0.605

CI, confidence interval; IRR, intensity rate ratio.

*P-*value obtained from likelihood ratio test.

**Figure 4:**
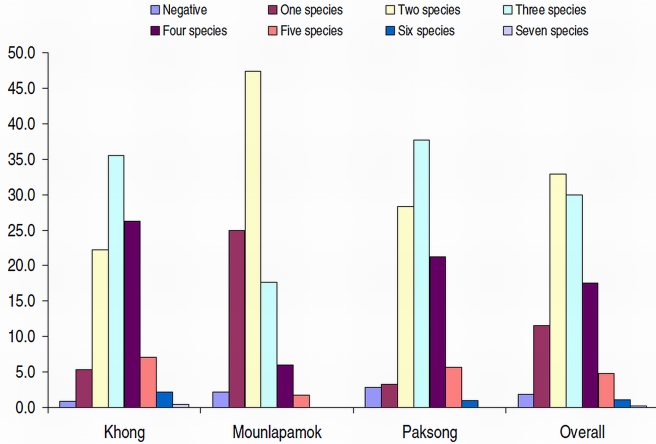
Multiparasitism as assesses by stool examination using two diagnostic methods, stratified by eco-epidemiological setting (n = 669).

Only 13 (1.9%) individuals were free of intestinal parasites. Mono-infections were observed in 77 individuals (11.5%). Hence, most of the study participants had a multiple species intestinal parasite infection: 32.9% were infected with two different parasites, 53.5% harbored 3–6 parasite species concurrently, and in one individual seven different parasites were observed. Over a third of the study participants living in Paksong and Khong districts were infected with three different parasite species concurrently and almost half of the surveyed Mounlapamok residents were concurrently infected with at least two parasite species (Figure 4).

### Parasite associations

Table 3 summarizes significant associations between different intestinal parasites. An *O. viverrini* infection showed a significant positive association with *S. mekongi* (odds ratio (OR)  = 5.09, 95% CI = 2.49–10.42), but negative association with both *A. lumbricoides* (OR = 0.05, 95% CI = 0.03–0.07) and *T. trichiura* (OR = 0.34, 95% CI = 0.20–0.58). Conversely, *S. mekongi* showed a significant positive association with an *O. viverrini* infection (OR = 5.64, 95% CI = 2.75–11.56). Moreover, there were significant positive associations between *S. mekongi* and *Echinostoma* spp. (OR = 3.19, 95% CI = 1.58–6.45) and between *S. mekongi* and two intestinal protozoa, namely *B. hominis* (OR = 2.19, 95% CI = 1.26–3.79) and *E. coli* (OR = 2.20, 95% CI = 1.01–4.83). An infection with hookworm was significantly associated with the other common soil-transmitted helminths (*A. lumbricoides* and *T. trichiura*) and *S. mekongi* (OR = 1.70, 95% CI = 1.04–2.79).

**Table 3:** Associations among parasite infections in Champasack province, Lao PDR (stepwise multiple logistic regression analyses).

	Association	OR (95% CI)	*P*-value
**Trematodes**			
*O. viverrini*	*S. mekongi*	5.09 (2.49–10.42)	<0.001
	*A. lumbricoides*	0.05 (0.03–0.07)	<0.001
	*T. trichiura*	0.34 (0.20–0.58)	<0.001
*S. mekongi*	*O. viverrini*	5.64 (2.75–11.56)	<0.001
	*Echinostoma* spp.	3.19 (1.58–6.45)	0.001
	*B. hominis*	2.19 (1.26–3.79)	0.005
	*E. coli*	2.20 (1.01–4.83)	0.049
*Echinostoma* spp.	*S. stercoralis*	2.07 (1.55–11.03)	0.005
**Nematodes**			
Hookworm	*S. mekongi*	1.70 (1.04–2.79)	0.034
	*A. lumbricoides*	10.64 (4.29–26.36)	<0.001
	*T. trichiura*	5.68 (2.32–13.87)	<0.001
*A. lumbricoides*	Hookworm	3.52 (3.64–19.05)	<0.001
	*E. coli*	3.51 (1.33–9.26)	0.011
	*T. trichiura*	2.52 (1.45–4.39)	0.001
	*O. viverrini*	0.05 (0.03–0.08)	<0.001
*T. trichiura*	*A. lumbricoides*	2.55 (1.50–4.34)	0.001
	Hookworm	2.38 (2.17–12.76)	<0.001
	*S. stercoralis*	4.59 (1.24–16.99)	0.022
	*Taenia* spp.	4.09 (1.61–10.36)	0.003
	*G. intestinalis*	2.61 (1.11–6.15)	0.028
	*O. viverrini*	0.08 (0.18–0.51)	<0.001
*E. vermicularis*	*E. coli*	4.03 (1.24–13.02)	0.020
*S. stercoralis*	*Echinostoma* spp.	8.60 (2.23–33.23)	0.002
	*T. trichiura*	4.29 (1.15–15.90)	0.030
**Cestodes**			
*Taenia* spp.	*T. trichiura*	3.40 (1.52–7.62)	0.003
*H. diminuta*	*A. lumbricoides*	19.69 (2.59–149.61)	0.004
**Intestinal protozoa**			
*B. hominis*	*S. mekongi*	2.19 (1.26–3.80)	0.005
	*E. coli*	3.78 (1.93–7.38)	<0.001
	*O. viverrini*	0.59 (0.35–0.99)	0.044
*E. coli*	*S. mekongi*	2.57 (1.23–5.37)	0.012
	*A. lumbricoides*	3.91 (1.94–7.90)	<0.001
	*E. vermicularis*	4.14 (1.26–13.61)	<0.019
	*B. hominis*	4.03 (2.05–7.92)	<0.001
*G. intestinalis*	*T. trichiura*	2.69 (1.21–6.00)	0.016

### Risk factors for parasitic infections

More than half of our fully compliant study participants (n = 345, 51.6%) reported to have consumed at least once raw fish dishes within 7 days prior to the interview. The habit of raw fish consumption was particularly frequent among the Lao-loum ethnic group (85.7%), and significantly less common among the Lao-theung ethnic group (14.3%; LRT = 98.04, P <0.001). Consumption of raw meat dishes was reported by 12.3% of our study population. Of those, 80.7% belonged to the Lao-loum and 19.3% to the Lao-theung ethnic group.

Table 4 shows the results from the multiple logistic regression analyses regarding associations between parasitic infections and risk factors, taking into account the random effect of households. Lao-loum ethnic groups were more likely to have an *O. viverrini* infection than Lao-theung ethnic groups (OR = 303.5, 95% CI = 134.2–686.6). The Lao-loum were at lower risks of hookworm (OR = 0.12, 95% CI = 0.07–0.23). Swimming (bathing) in the Mekong River was a key risk factor for acquiring a *S. mekongi* infection. Infections with *A. lumbricoides* was more common in poorer population segments (most poor: OR = 3.53, 95% CI = 1.47–8.47). Consuming of raw or insufficiently cooked food was a risk factor for multiparasitism in our study population (OR = 2.74, 95% CI = 1.44–5.20).

**Table 4:** Associations between parasitic infections and risk factors in Champasack (random household effect included, n = 669).

Indicators	Crude OR (95%CI)	*P*-value	Adjusted OR (95% CI)	*P*-value
***O. viverrini***				
Age group (in year)				
< 5	1.00	- ---	1.00	- ---
6–15	5.64 (2.10–15.13)		3.99 (1.93–8.24)	
16–30	19.49 (5.53–68.67)		17.5 (5.70–53.68)	
31–55	19.93 (5.97–66.51)		12.30 (4.68–32.29)	
>55	49.74 (8.00–309.04)	<0.001	13.96 (3.32–58.75)	<0.001
Ethnic groups				
Not Laoloum	1.00		1.00	- ---
Laoloum	154.9 (81.20–295.70)	<0.001	303.45 (134.20–686.63)	<0.001
***S. mekongi***				
Daily bathing in Mekhong River				
No	1.00		1.00	- ---
Yes	19.50 (8.87–42.87)	<0.001	3.20 (1.84–5.83)	<0.001
**Hookworm**				
Age group (in year)				
< 5	1.00	- ---	1.00	- ---
6–15	2.32 (1.24–4.34)		2.21 (1.28–3.80)	
16–30	2.51 (1.23–5.14)		2.22 (1.16–4.23)	
31–55	2.39 (1.24–4.62)		2.10 (1.18–3.72)	0.011
>55	2.26 (1.01–5.50)	0.040	N.A.	N.S.
Ethnic groups				
Not Laoloum	1.00	- ---	1.00	- ---
Laoloum	0.11 (0.05–0.23)	<0.001	0.12 (0.07–0.23)	<0.001
***A. lumbricoides***				
Socio-economic status				
Least poor	1.00	- ---	1.00	- ---
Less poor	0.35 (0.06–2.10)		N.A.	
Poor	1.27 (0.26–6.11)		N.A.	
Very poor	50.14 (10.39–241.97)		N.A.	
Most poor	226.73 (41.64–434.43)	<0.001	3.53 (1.47–8.47)	0.010
**Multiple infections ≥2 species**				
Report of eating any raw foodstuffs a week prior to survey (e.g., meat, fish, and vegetables)				
No	1.00		1.00	- ---
Yes	2.12 (1.15–3.90)	0.021	2.74 (1.44–5.20)	0.002

CI, confidence interval; N.S., not significant; N.A., not applicable; OR, odds ratio.

## Discussion

Helminth infections are widespread in Lao PDR and the Great Mekong sub-region in general. *S. mekongi, O. viverrini*, various MIFs and soil-transmitted helminths are prevalent and there is extensive geographical overlap of various helminth infections [9], [21], [22], [32], [33]. However, there is a paucity of high-quality data to elucidate the extent of multiparasitism and underlying risk factors [23], [34]. We conducted a cross-sectional study in three distinct eco-epidemiological settings of Champasack province situated in the southern part of Lao PDR. We employed a rigorous diagnostic approach, i.e., at least two stool samples were collected over consecutive days and examined by the KK method, supplemented with a FECT performed on one of these stool samples.

Our data confirm that multiple species intestinal parasite infections are the norm rather than the exception; indeed more than 4 out of 5 study participants with complete data records harbored at least two different species concurrently, and several intestinal parasite species were found at high prevalence rates. Worryingly, *O. viverrini* infections were found in over 90% of the study subjects in the two low-land settings (Khong and Mounlapamok districts). In Khong district, additionally, we found a high *S. mekongi* infection prevalence (68.0%). Soil-transmitted helminths were common in the highland of Paksong district; the overall prevalence for hookworm, *A. lumbricoide*s and *T. richiura* were 94.8%, 85.9% and 55.7%, respectively. On the other hand, intestinal protozoa infections (*B. hominis, E. coli, G. intestinalis* and *E. nana*) were far less prevalent (<14.0%).

Limitations of our study are as follows. First, although we employed a rigorous diagnostic approach, the ‘true’ extent of multiparasitism is still underestimated. The diagnostic techniques used in our study only have a low sensitivity for the detection of certain parasite species (e.g., *Strongyloides stercoralis* and MIF) or are inadequate for other endemic parasitic infections such as malaria. Second, we did not differentiate eggs of *O. viverrini* from those of MIF. Eggs of *O. viverrini* and MIF are similar in size and shape, and hence it is exceedingly difficult to differentiate them under a microscope. Therefore among those study participants declared *O. viverrini*-positive, some might actually be infected with MIF, since many species of MIF are also endemic in Lao PDR [16], [22], [35]. In our own preceding work, we found that multiple trematode species infections indeed are common. For example, among 97 individuals with heavy *Opisthorchis* infections who were purged, 81.4% of the participants were multi-parasitized. *O. viverrini* was the most common trematode (97.9%), followed by *Haplorchis taichui* (78.4%). Other small intestinal fluke species were less common [22]. In studies carried out elsewhere in Lao PDR, it was also found that *O. viverrini* is the predominant trematode species [16], [17]. Third, it cannot be ruled out that some of the diagnosed hookworm eggs were actually infections with *Trichostrongylus* spp. The latter parasite has been found in Lao PDR with notable prevalence rates [36].

Highest infection intensities of *A. lumbricoides* and *T. trichiura* were observed in pre-schoolers (aged ≤5 years), whereas the peak infection intensities of *S. mekongi* and hookworm were observed in school-aged children (age: 6–15 years). Adults aged above 55 years showed highest *O. viverrini* infection intensity rate ratios.

The high prevalence of *S. mekongi* observed in Khong district must be emphasized. This finding suggests that schistosomiasis is still a public health concern in southern Lao PDR. Once schistosomiasis had been recognized as a major public health problem in southern Lao PDR and Cambodia in the early 1980s and early 1990s, respectively [10], [37]–[39], community-based control programs were launched. The aim of these control programs was to reduce schistosome-related morbidity. Large-scale administration of praziquantel was endorsed as the strategy of choice [9], [10], [38]. Multiple rounds of praziquantel reduced the prevalence of *S. mekongi* in the endemic areas to very low levels in 1998 (2.1% in Khong district and 0.4% in Mounlapamok district) and was considered a successful public health control program [9], [10]. However, interruption of chemotherapy-based morbidity control in face of inadequate sanitation, lack of clean water, and continued human water contacts are at the root of rapid re-infection and re-emergence of schistosomiasis. In 2006, chemotherapy-based control has been re-established. Failure to improve access to clean water and adequate sanitation will render truly sustainable schistosomiasis control a distant goal. In 2007 in our study villages of Khong district, only 14.5% of the households possessed latrines and 76.0% reported daily use of the Mekong River for bathing (K. Phongluxa, personal communication). Hence, there is also a need for more vigorous health education to avoid risky water contacts as a means of lowering the transmission of schistosomiasis and to thoroughly cook fish and other aquatic products to break the transmission cycle of opisthorchiasis and other food-borne trematode infections.

Our findings underscore that intestinal multiparasitism is common throughout Champasack province. The same observations have been made in other parts of Lao PDR [15], [16], [22] and neighboring countries such as Vietnam [32], [33], [40] and southern P.R. China [41]. Indeed, multiparasitism is the rule rather than exception in the developing world [23], [42], [43], and hence it is surprising that the topic has received only token attention [44]. Our data showed that *O. viverrini* and hookworm co-infections were highly prevalent in the plain area of Khong and Mounlapamok districts, whilst multiple species soil-transmitted helminth infections were common among the study participants in the highlands of Paksong district.

From a clinical point of view, co-infection of *S. mekongi* and *O. viverrini* is of particular concern. Indeed, an infection with *O. viverrini* leads to severe clinical manifestations such as hepato-biliary pathologies, including hepatomegaly, obstructive jaundice, gallbladder stones, cholecystitis and cholangitis and, most importantly, the development of a fatal bile duct cancer (cholangiocarcinoma) [11], [13], [45]. Chronic infection with *S. mekongi* contributes to a formation of hepatomegaly, periportal fibrosis, and portal hypertension [37], [46]–[48]. Co-infections of these two trematodes might further aggravate the host-organ pathology, especially the liver.

Another interesting finding of our study was the significant association observed between *S. mekongi* and hookworm. Interestingly, previous studies carried out in Côte d'Ivoire found a significant association between *S. mansoni* and hookworm [42], [43], [49]. Whilst the distribution of single species infection and co-infections have been mapped [50] and risk factors elucidated, there is a lack of epidemiologic investigations focusing on symptoms and morbidities due to co-infections.

Soil-transmitted helminths were also found to be highly prevalent in the present study, particularly among those living in the highlands of Paksong district. An infection with soil-transmitted helminths can lead to nutritional deficiencies and may impair growth and cognitive development in children [50]–[53]. It is widely acknowledged that children aged below 5 years are at a high risk of mortality in developing countries [54]. Although the causes of death are multi-factorial, malnutrition is a key factor and parasites contribute to a substantial fraction of this under-nourishment [54], [55]. In Lao PDR, a national deworming program is currently being implemented at the school level in collaboration with the MoH and the Ministry of Education [56]. However, preschool-aged children are currently not part of the project. Our findings of high prevalence and infection intensity of soil-transmitted helminths among preschool–aged children should be considered for future control activities. It is conceivable that including under-5 year-old children into the deworming program might improve their health status.

Epidemiologic studies have shown that prevalence and intensity of several parasitic infections are governed by behavioral, socioeconomic, and environmental characteristics [31], [34]. In the current study, we observed that the consumption of raw or insufficient cooked fish through traditional dishes (i.e., “Lap-pa” and “Koy-pa”) was commonly practiced among the Lao-loum ethnic groups. This is the most likely explanation why the Lao-loum were at a significantly higher risk of *O. viverrini* infection than Lao-theung. This behavioral trait is known to be a potential risk for acquiring *O. viverrini* and other fish-borne trematode infections [15], [57], particularly in an area where several food-borne trematodes co-exist, such as Lao PDR [16]–[18], [22]. It is also important to note that intestinal parasites varied according to people's socioeconomic status. Interestingly, *S. mekongi* was significantly more prevalent among better-off study participants who live along the lower Mekong islands. On the other hand, as expected, the highest prevalence of soil-transmitted helminths, particularly *A. lumbricoides* and *T. trichiura,* were observed among the poorest living in the highlands. The latter finding is in line with observations reported from southern P.R. China [31] and other parts of the developing world [58]. People belonging to the poorest wealth quintiles are at higher risk of infection with soil-transmitted helminths.

Finally, we found a low prevalence of intestinal protozoa in our study cohort. These findings support the previous observations, which have shown low prevalence of pathogenic intestinal protozoa in Southeast Asia [23], [41], [59].

We conclude that multiparasitism is the rule in different eco-epidemiological settings of Champasack province, and most likely elsewhere in Lao PDR. The extent of multiparasitism and the high infection prevalence and intensity with a host of intestinal parasites, most importantly *S. mekongi* and *O. viverrini* are a public health problem. Consequently, a chemotherapy-based morbidity control program should be re-implemented without delay. To consolidate progress and ascertain long-term sustainability, other control measures such as health education, improving access to clean water and sanitation in an intersectoral fashion must be considered.

## Supporting Information

Informed consent form (0.04 MB PDF)

STROBE checklist (0.09 MB DOC)
