# Supplementary material for: Helminth and Intestinal Protozoa Infections, Multiparasitism and Risk Factors in Champasack Province, Lao People's Democratic Republic
Source: PLoS Negl Trop Dis. 2011 Apr 12;5(4):e1037. doi: 10.1371/journal.pntd.0001037 (PMC3075221; doi:10.1371/journal.pntd.0001037)
Supplement: Informed consent form — (0.04 MB PDF) [file pntd.0001037.s001.pdf]

1 Information and Consent Form for the Household Head

Parasite infection and disease in Champassak Province, Laos

Parasite infections are very common in Laos. We are studying many villages for worm infection and we are providing treatment. This research is by Lao's National Institute of Public Health (NIPH) & the Swiss Tropical Institute and funding is from a Swiss Foundation.

Study aims are to find out the level of parasite infection and the diseases it causes inside the body.

Procedure: We ask you and every member of your Household aged > 6 months to submit **1 Stool Samples to look for worm eggs**. **Please do not mix containers. Each container is labeled for each person. Bring the stool sample to the Research House and** A doctor will perform a **Physical Exam** for you. We will take a drop of blood from a **Fingerprick** to check for anaemia or low blood level. **You will be given the results**. **If we find parasites we will observe your Treatment,** usually 1 or 2 drugs (Praziquantel, Albendazole). There is **no charge** for this treatment.

The **benefits** to participate will be learning information about your health and treatment for parasite infections. **Participation is voluntary** which means you can leave the study at any time. There are **no costs** to you or your family in this study. The **risk** to participate is very low. Any unusual symptoms or other concerns should be reported to us. **You have full confidentiality & privacy.** The data may be published but no names will be released.

Contact Information: Any concerns or questions, please direct them immediately to the field team or call: Dr. Somphou Sayasone +856-20 567-9603. Additional numbers are: Dr. Kongsap Akkhavong +856-20 550-9725 (Laos) and Dr. Peter Odermatt (Switzerland) +4161 284 82 14.

I ______________________________ have read or discussed all of the above. My questions were fully answered. I am aware of my rights to freely participate in this study. **I sign on behalf of myself and my household members.**

______________________	______________________
Signature of Household Head	Signature of Research Interviewer
______________________	______________________
	Signature of Witness
______________________	______________________
Date	

2 Line Listing of Household Members

Village Name:
District Name:
Province: Champassak
Today's Date:

Village No. ☐☐ Household Registration No. ☐☐☐

INDIVID NO.	Family Name, First Name (in Lao and in English)	Age years	Sex M/F	Relation to HHold Head **	Mother's Individ No. (< 15 yrs)	Study Protocol: Check √ when completed							Special Notes (eg. Absent on day of study Venipuncture, Purging, reason for incomplete study, etc)
						Individual	Physical	US	Stool			Finger	
									1	2	3		
01	HEAD			------									☐ Household Quest.
02													
03													
04													
05													
06													
07													
08													
09													
10													
11													
12													
13													
14													
15													
16													
17													
18													
19													
20													

Ask: Who is the Household Head (Number 01)? Who is the spouse (02)? List ALL people who usually share meals AND sleep here (even if absent from house). **Relation to Head: **1** =husband, **2**=wife, **3**=daughter, **4**=son, **5**=mother, **6**=father, **7**=brother, **8**=sister, **9**=uncle*,* **10**=aunt, **11**=cousin. **12**=Specify if other. If any Household member is not erviewed or absent from house or has an incomplete Study Protocol please describe reason.
